# Viral hepatitis and *Treponema pallidum* prevalence in persons who underwent premarital blood tests in Argentina

**DOI:** 10.1038/s41598-019-45891-9

**Published:** 2019-07-03

**Authors:** Patricia Angeleri, Valeria Levite, Gabriela Vidiella, Joaquín Solari, Emma Coronel, Dan Adaszko, Ariel Adaszko, Cecilia Moyano, Diosnel Bouchet, Héctor Cuello, Viviana Molfese, Rosario Skarzauskas, Marcelo Vila, Carlos Falistocco, Maria A. Pando

**Affiliations:** 1 Dirección de SIDA y ETS. Ministerio de Salud de la Nación, Ciudad Autónoma de Buenos Aires, Argentina; 2 Programa Provincial de ETS y Sida, Santa Fe, Argentina; 3 Programa Provincial de Lucha contra el VIH-sida y ETS, Córdoba, Argentina; 4 Programa Provincial de Hepatitis Virales, Mendoza, Argentina; 5 Programa Provincial de VIH-sida, ITS y Hepatitis Virales, Buenos Aires, Argentina; 6 Organización Panamericana de la Salud/Organización Mundial de la Salud, Ciudad Autónoma de Buenos Aires, Argentina; 7 CONICET- Universidad de Buenos Aires. Instituto de Investigaciones Biomédicas en Retrovirus y SIDA (INBIRS), Ciudad Autónoma de Buenos Aires, Argentina

**Keywords:** Infectious-disease diagnostics, Population screening, Population screening, Infectious-disease diagnostics

## Abstract

The objective of this study was to estimate the prevalence of different serological markers of hepatitis A, B and C viruses and *Treponema pallidum* among the adult population of Argentina. To achieve this, adults who attended health services for premarital exams (which are mandatory and includes screening for syphilis) were recruited. A cross-sectional study was designed with a cluster sampling strategy. Couples who attended selected health services for premarital screening between 2013 and 2014 in Buenos Aires, Cordoba, Mendoza and Santa Fe provinces were included. A total of 3833 individuals were recruited. Anti-HAV prevalence was 63.9%, anti-HCV 0.3%, anti-HBc (without HBsAg) 1.9%, HBsAg 0.3%, and *T pallidum* 0.8%. Anti-HAV was higher among older participants, foreigners and those from the lower strata. HBV increased with age and was higher among foreigners and those with lower formal educational level. Anti-HCV frequency increased with age. Premarital screening of viral hepatitis could constitute an instance of diagnosis, vaccination and inclusion in care of those in need. Results from this study will allow the national hepatitis programs to design public policies in order to diminish the impact of these infections on the population.

## Introduction

Viral hepatitis is a major public health problem that affects 400 million people worldwide and is responsible for 1.4 million deaths per year including both, acute infection and hepatitis-related liver cancer and cirrhosis. Most of those deaths (95%) are attributable to hepatitis B and C. The five hepatitis viruses described (A, B, C, D and E) are very different; they are characterized by different transmission routes and probabilities, affecting diverse groups of the population, existing differential prophylaxis options and in consequence, resulting in distinct health outcomes^1^. Hepatitis A virus (HAV) and Hepatitis E virus (HEV) are both enterically transmitted acute infections. Viral infections occur mostly by person-to-person transmission and through consumption of contaminated water or food. Therefore, prevalence is strongly associated with socioeconomic development. Vaccination against HAV has effectively reduced the infection burden. Transmission of both, HAV and HEV, can be prevented by improving sanitation and access to safe water and food. Hepatitis B virus (HBV) and Hepatitis C virus (HCV) are transmitted through contact with infected blood or semen. More than 95% of adults with hepatitis B infection resolve it during the acute phase, however 95% of neonates and 20–30% of children between 1–5 years of age progress to chronic infection. The implementation of universal HBV vaccination has resulted in a decrease in prevalence in many parts of the world. As regards HCV, approximately 80% of acute hepatitis C cases progress to chronic infection, and 10–20% of these will develop chronic liver disease complications such as cirrhosis and/or hepatocellular carcinoma^1,2^.

Viral hepatitis infections are mostly diagnosed by testing serological markers. The diagnosis of acute HAV infection is made by detecting the presence of anti-HAV IgM antibodies. For epidemiological issues, total anti-HAV antibodies (IgM and IgG) can also be tested. For HBV, the diagnosis testing should include several serological markers: HBsAg, anti-HBs, HBeAg, anti-HBe, and anti-HBc IgM and IgG. However, HBsAg is the serological hallmark of infection due to the fact that HBsAg can be detected within 1 to 10 weeks after infection, and persistence of this marker for more than 6 months suggests chronic infection. Previous HBV infection is usually detected by the presence of anti-HBc. HCV screening is performed by an antibody test, and when the anti-HCV antibody test is positive, current infection should be confirmed by HCV RNA test^2^.

The World Health Organization also estimates that only 1 in 20 individuals with hepatitis know their status and that 1 in 100 of those infected are on treatment^1^. These epidemiological data clearly reveal the need to improve the tools and approaches currently available, like extended hepatitis A and B vaccination and hepatitis C treatment that can attain cure rates of over 90%. To achieve these objectives, WHO adopted a new strategy: the “Prevention and Control of Viral Hepatitis Infection: Framework for Global Action” aimed at eliminating viral hepatitis as one of the major public health threats by 2030.

In line with international strategies, a national program on viral hepatitis control was implemented in Argentina in 2012 (*Programa Nacional de Control de las Hepatitis Virales, Dirección de SIDA y ETS* (*DSyETS*), *Ministerio de Salud y Desarrollo Social de la Nación*). The main objectives of this program are to warrant prevention, diagnosis and treatment of viral hepatitis in order to decrease the incidence of infections. Currently, Argentina has provided access to hepatitis A and B vaccines since 2005, for newborns/children since 2000, and for adults since 2012, respectively. However, due to their recent universal adoption, most adult populations are not immunized. Regarding hepatitis C, Argentina has been providing treatment to all patients regardless of liver fibrosis since December 2017. Epidemiological studies on viral hepatitis conducted in Argentina have mostly been focused on most at-risk groups. These studies revealed a high HBV prevalence among men who have sex with men (23–37%), male-to-female transgenders (40%), drug users (injecting, 42% and non-injecting, 9%), as well as female sex workers (14%). High prevalence of hepatitis C virus was also identified in the same groups (1.9–7.5% among MSM, 4.5% among male-to-female transgenders, 54.6% among injecting drug users, 7.5% among non-injecting drug users, and 4.3% among female sex workers)^3–7^. However, no previous studies have investigated the prevalence of HCV among less exposed groups.

Syphilis is a sexually transmitted bacterial infection caused by *Treponema pallidum subspecies pallidum (T. pallidum*). Even when syphilis can be efficiently diagnosed and treated, it continues to be an important health problem. The global syphilis prevalence has been estimated at 0.5% (0.4–0.6%) and the regional values range from 0.2% to 1.8% with the African Region having the highest incidence rate followed by the region of the Americas.^8,9^. The global prevalence of syphilis in Argentina is unknown, however, previous studies conducted by our group found a high prevalence among men who have sex with men (MSM) (20.5%), female sex workers (22.4%) and female transgender sex workers (50%)^4,5,10^.

The main goal of this study was to estimate the prevalence of different serological markers of hepatitis A, B and C viruses and *Treponema pallidum* among the adult population of Argentina. To achieve this objective, adults who attended health services aimed at premarital exams were studied. Premarital exam, which only includes screening for syphilis, is mandatory and free of charge in Argentina. The study population included male and female adult individuals from all socioeconomic and sociocultural strata.

## Methods

### Study population

We conducted a cross-sectional study using a cluster sampling strategy. Adult individuals who attended health services for premarital exams (which includes screening for syphilis) were included in the study. Recruitment was performed in Buenos Aires, Greater Cordoba, Río Cuarto, Greater Mendoza, Greater Santa Fe, and Rosario from September 2013 to October 2014.

### Ethics statement

International and national ethical guidelines for biomedical research involving human subjects were followed. This research study was approved by a local Institutional Review Board (IRB) (*Comité Independiente de Etica en Investigación, Nexo AC Buenos Aires, Argentina*), and conducted in compliance with all federal regulations governing the protection of human subjects. All potential participants signed a written informed consent prior to entering the study.

### Sampling methodology

Sampling places were selected through cluster sampling considering places with more than 5000 marriages per year according to the last census (2010). Then, we considered the logistic possibilities in each city. Finally, the following provinces, cities and hospitals were selected for sampling: Buenos Aires province: Melchor Romero (Hospital Interzonal de Agudos y Crónicos Dr. Alejandro Korn), La Plata (Hospital Interzonal Especializado de Agudos y Crónicos San Juan de Dios, and Hospital Interzonal General de Agudos, General San Martín), Lanus (Hospital Interzonal General de Agudos Evita), and Florencio Varela (Hospital Zonal General de Agudos Mi Pueblo); Córdoba Province: Córdoba City (Hospital Rawson, and Centro Municipal de la Ciudad de Córdoba), and Rio Cuarto (Centro Municipal de la Ciudad de Río Cuarto); Mendoza Province: Mendoza City (Hospital Central and Centro de Salud N°2), and Guaymallén (Centro de Salud N°16); and Santa Fe Province: Santa Fe City (Hospital Dr. José María Cullen), and Rosario (Hospital Provincial, and Centro de Especialidades Médicas Ambulatorias de Rosario (CEMAR)).

### Sample collection and diagnosis of viral hepatitis and Treponema pallidum

Anticoagulated blood was collected for the determination of viral hepatitis A, B and C as well as *T pallidum* infection. Diagnosis of *T pallidum* infection was conducted using quantitative VDRL (VDRL, Wiener Laboratorios, SAIC, Rosario, Argentina). Markers of hepatitis A, B and C infection were determined using ELISA (BIOELISA HAV 96 TESTS, BIOELISA HBsAg 3.0 96 TESTS, BIOELISA ANTI-HBC 96 TESTS, and BIOELISA HCV 4.0 96 TESTS, Biokit, Werfen, Barcelona). Anti-HBc presence without HBsAg detection was considered as an indicative of a previous exposure to HBV, and HBsAg presence was considered as a marker of present infection.

All participants obtained their lab results during medical consultation and those who needed it were referred to local hospitals for medical care and treatment.

### Statistical analysis

All data were included in a database and analyzed using SPSS (IBM Corp. Released 2013. IBM SPSS Statistics for Windows, Version 22.0. Armonk, NY: IBM Corp.). In all the analyses, the sample was weighted. Continuous variables were described using medians and interquartile ranges (IQRs), and categorical data, using counts and percentages. For seroprevalences, ninety-five percent confidence intervals (95% CI) were estimated using exact binomial formula. Bivariate analyses were performed using Chi square test or Fisher’s exact test according to sample size. All p-values were two-sided and considered to be statistically significant when p < 0.05.

## Results

### Characteristics of the study group

A total of 3833 participants (1940 women, 50.6% and 1893 men, 49.4%) were recruited between September 2013 and October 2014. Table 1 shows demographic characteristics of the participants classified by gender. Briefly, the median age of the participants was 30 years (IQR = 26–36, range = 18–84); most of them (94.2%) were born in Argentina and 68.3% completed high school level. In relation to their housing situation, 8.0% reported to live in precarious places, like shantytowns. A total of 76.6% individuals reported having a job position. However, only 76.4% of them make pension contributions. Regarding medical insurance, 66.5% of all the study population reported having it, either the job-based (55.0%) or pre-paid (11.5%). Considering all socioeconomic variables, samples were stratified into two strata: middle strata (66.6%) and lower strata (33.4%). No significant differences were observed between men and women except for employment status, pension contributions and health insurance, where a higher frequency was observed in men.

**Table 1:** Demographic characteristics of 3833 participants recruited in Argentina between September 2013 and October 2014.

	Men N = 1893% (n)	Women N = 1940% (n)	p value
**Province**			
Buenos Aires	38.5 (735)	38.3 (738)	0.994
Córdoba	20.0 (383)	19.9 (383)	
Santa Fe	24.4 (466)	24.8 (477)	
Mendoza	17.1 (327)	17.0 (327)	
**Age**			
**Median, years (IQR)**	30.0 (27–36)	30.0 (26–35.9)	0.561
<30 years old	50.7 (968)	51.7 (994)	
≥30 years old	49.3 (942)	48.3 (930)	
**Nationality**			
Argentinean	94.6 (1806)	93.9 (1807)	0.370
Foreigners	5.4 (104)	6.1 (118)	
**Housing situation**			
House/apartment	93.8 (1792)	93.1 (1792)	0.398
Poor housing	6.2 (119)	6.9 (133)	
**Formal educational Level**			
Completed high school	68.7 (1312)	68.8 (1325)	0.917
Incomplete high school	31.3 (599)	31.2 (600)	
**Employment**			
Yes	92.1 (1760)	59.6 (1148)	<**0.001**
No	7.9 (150)	40.4 (777)	
**Pension contributions (n = 2936)**			
Yes	78.2 (1377)	74.5 (855)	**0.020**
No	21.8 (383)	25.5 (293)	
**Health insurance**			
Yes	70.7 (1351)	61.1 (1177)	**0.000**
No	29.3 (560)	38.9 (748)	
**Strata**			
Middle	66.0 (1261)	65.3 (1257)	0.658
Lower	34.0 (649)	34.7 (667)	

### Prevalence of viral hepatitis and T. pallidum

A total of 3833 participants were studied for anti-HAV, anti-HBc, HBsAg, anti-HCV and *T pallidum* infections. As per Table 2, anti-HAV prevalence was 63.9% (95%CI 62.4–65.6), total anti-HBc 2.0% (95%CI 1.53–2.46), anti-HBc (without HBsAg) 1.91% (95%CI 1.47–2.35), HBsAg 0.27% (95%CI 0.10–0.43), anti-HCV 0.26% (95%CI 0.10–0.43), and *T pallidum* 0.74% (95%CI 0.47–1.01). No significant differences were observed between men and women. No cases of anti-HBc/anti-HCV or HBsAg/anti-HCV co-infection were identified in this cohort.

**Table 2:** Prevalence of viral hepatitis and syphilis among 3833 participants recruited in Argentina from September 2013 to October 2014.

Prevalence	Total N = 3833% (n)
IgG anti-HAV	63.9 (2442)
IgG anti-HCV	0.3 (10)
Previous exposure to HBV HBsAg−anti-HBc+	1.9 (71)
Acute or chronic HBV infection HBsAg+anti-HBc+/−	0.3 (10)
*Treponema pallidum*	0.8 (29)

Viral hepatitis and *T. pallidum* prevalence were analyzed according to stratification among cities (Table 3). *T. pallidum* was significantly more frequent among participants from Buenos Aires (p = 0.008) and Santa Fe (p = 0.012) compared with Cordoba, and anti-HBc (without HBsAg) was significantly higher in Buenos Aires compared with other places (3.5%, p < 0.001).

**Table 3:** Prevalence of viral hepatitis and syphilis among 3833 participants recruited in Argentina from September 2013 to October 2014. Stratification according to province.

Prevalence	Total N = 3833% (n)	Buenos Aires N = 1457% (n)	Cordoba N = 765% (n)	Mendoza N = 650% (n)	Santa Fe N = 932% (n)	p value
IgG anti-HAV	63.9 (2442)	65.3 (957)	62.9 (481)	64.4 (418)	62.4 (587)	0.193
IgG anti-HCV	0.3 (10)	0.1 (1)	0.5 (4)	0.3 (2)	0.3 (3)	0.271
Previous exposure to HBV (HBsAg−, anti-HBc+)	1.9 (71)	3.5 (51)	0.5 (4)	1.4 (9)	0.7 (6)	<0.001
Acute or chronic HBV infection (HBsAg+, anti-HBc+/−)	0.3 (10)	0.3 (5)	0.3 (2)	0.5 (3)	0.1 (1)	0.535
Previous exposure, acute or chronic HBV infection	2.2 (81)	3.7 (55)	0.9 (7)	1.8 (12)	0.9 (7)	<0.001
*Treponema pallidum*	0.8 (29)	0.8 (12)	0.1 (1)	0.8 (5)	1.0 (9)	0.579

### Demographics and risk factor analysis

In relation to HAV, anti-HAV prevalence increased with age (p < 0.001). A higher frequency was also detected among foreigners (80.2% vs. 62.9%, p < 0.001) and those from the lower strata (80.5% vs. 55.2%, p < 0.001). The latter association reflects the higher frequency of anti-HAV observed among those living in precarious places (80.1% vs. 62.8%, p < 0.001), having lower formal educational level (80.3% vs. 56.5%, p < 0.001), without employment (74.3% vs. 60.6%, p < 0.001), without pension contributions (72.5% vs. 57.0%, p < 0.001), and without social security (78.2% vs. 56.5%, p < 0.001). With regard to HBV, Anti-HBc and/or HBsAg presence (indicative of previous exposure to the virus and present infection, respectively) also increased with age (p < 0.001). Higher frequency of infection was also detected among foreigners (mostly from Peru, Paraguay and Bolivia) as compared to Argentineans (6.8% vs. 1.9%, p < 0.001) and those with lower formal educational levels (3.0% vs. 1.8%, p = 0.038). As well as anti-HAV and Anti-HBc and/or HBsAg, anti-HCV frequency increased with age (p = 0.007). However, no other variables were associated with infection. A significantly higher frequency of *T. pallidum* was found among those who had not completed high school compared to those who had (1.3% vs. 0.5%, p = 0.017) (Table 4).

**Table 4:** Demographics and risk factor analysis for viral hepatitis and syphilis of 3833 participants recruited in Argentina between September 2013 and October 2014.

	HAV % (p value)	HBV % (p value)	HCV % (p value)	T. pallidum % (p value)
**Age**				
<30 years old	**59.0**	**1.2**	**0.1**	0.7
≥30 years old	**69.2**	**3.1**	**0.5**	1.1
	**(<0.001)**	**(<0.001)**	**(0.010)**	(0.221)
**Nationality**				
Argentinean	**62.9**	**1.9**	0.3	0.8
Foreigners	**80.2**	**6.8**	0	1.8
	**(<0.001)**	**(<0.001)**	(1.0)	(0.121)
**Housing situation**				
House/apartment	**62.8**	2.2	0.3	0.9
Poor housing	**80.1**	1.7	0	1.2
	**(**<**0.001)**	(0.817)	(1.0)	(0.487)
**Formal educational Level**				
Completed high school	**56.5**	**1.8**	0.3	**0.5**
Incomplete high school	**80.3**	**3.0**	0.3	**1.3**
	**(<0.001)**	**(0.038)**	(1.0)	**(0.017)**
**Employment**				
Yes	**60.6**	2.2	0.2	0.8
No	**74.3**	2.0	0.4	1.1
	**(**<**0.001)**	(0.793)	(0.268)	(0.415)
**Pension contributions**				
Yes	**57.0**	2.2	0.3	0.9
No	**72.5**	2.5	0	0.4
	**(**<**0.001)**	(0.652)	(0.347)	(0.330)
**Health insurance**				
Yes	**56.5**	2.1	0.2	0.8
No	**78.2**	2.4	0.3	1.2
	**(**<**0.001)**	(0.554)	(0.743)	(0.209)
**Strata**				
Middle	**55.2**	1.9	0.3	0.7
Lower	**80.5**	2.7	0.2	1.2
	**(**<**0.001)**	(0.095)	(1.0)	(0.145)

## Discussion

Viral hepatitis infections are regarded as one of the main global health problems caused by five unrelated viruses, with HAV, HBV and HCV being of utmost epidemiological importance. Despite the fact that HAV and HBV vaccines have been available for many years and that there is strong evidence of a decrease in prevalence rates, these infections still constitute a health problem worldwide. In the case of HCV, new treatments based on direct-acting antivirals (DAAs) have revolutionized the cure of chronic HCV infection with rates above 90%. Even when vaccines and treatments are making progress on viral hepatitis eradication as a public health problem, there are still many challenges to face. The Global Health Sector Strategy (GHSS) on viral hepatitis 2016–2021 calls for the elimination of viral hepatitis as a public health threat by 2030 through the reduction of new infections by 90% and mortality by 65% compared with the 2015 baseline^11^.

In order to achieve these goals, a national program on viral hepatitis control was created in Argentina in 2012. In this context, this research study was designed to contribute to the information on the situation of viral hepatitis in the adult population. To obtain a representative sample of adults we decided to study individuals who attended public health services aimed at premarital exams (which includes screening for syphilis only by law). This strategy has been used in other countries in order to obtain more accurate epidemiological data regarding viral hepatitis carrier rates in the general population^12^. The advantage of this population is that it comprises men and women with different educational levels and socio economic strata, due to the fact that in Argentina most individuals attend public hospitals for premarital examinations.

A single-dose hepatitis A virus vaccination was implemented in Argentina aimed at children of 12 months of age in 2005. After that, there was an abrupt decline in the incidence of HAV infection from 66.5 to 7.9/100.000, representing a decrease in 88.1%. A dramatic decline in fulminant hepatic failure (FHF) and liver transplantation (LT) cases has also been observed^13^. Due to the fact that the HAV vaccine has been recently introduced only for children, we assume that the adults included in our study were mostly not vaccinated. Therefore, the antibodies detected in our cohort are commonly related to previous infections. In line with previous studies, a high anti-HAV prevalence rate (64%) was found, which was even higher among older participants and those with lower socioeconomic levels. The increase in prevalence with age was described previously and it was associated with the time of exposure to the virus as well as the fact that older individuals could have been exposed to worse sanitary conditions^14^. The high frequency found among those with low socioeconomic levels could probably be related to the ingestion of contaminated water and/or food. Previous studies clearly established that the lack of access to safe drinking water and sanitary facilities is related to the increase in anti-HAV prevalence rates. Several research studies performed in Argentina demonstrated the circulation of HAV in wastewater and river samples from different regions^15,16^. These results highlight the need to improve sanitation and the access to clean water, which along with mass vaccination will contribute to reducing viral circulation and infection^17^.

The latest available data on the prevalence of HBV and HCV in Argentina is based mainly on blood donors and specific at-risk groups^3–7,18^. Studies performed among blood donors revealed that HBV and HCV prevalence have significantly decreased over the past years. Considering a ten-year period (2004–2014), these prevalence rates decreased from 0.41 to 0.20% for HBsAg (p < 0.001), 2.31 to 1.38% for anti-HBc (p < 0.001) and 0.74 to 0.39% for anti-HCV (p < 0.001). The decrease in prevalence rates could be attributed to several factors, like the implementation of voluntary/repeated donors instead of family/replacement donors (as the former have lower prevalence rates than the latter), the improved prevention methods used (e.g. HBV vaccination), changes in risk factors (e.g. a decline in injecting drug use), and the progress made in the specificity of screening tests^18^. In general, trends in blood donors provide an estimation of the trend in the general population, even when blood bank data may underestimate real prevalence rates. In fact, when comparing these prevalence data with the results obtained in our study population (both with similar age range; 18–84 in our study and 18–65 in blood banks), a higher frequency of anti-HBc in our cohort was found (1.9 vs. 1.38, p = 0.016). However, no significant differences were observed in HBsAg or anti-HCV prevalence rates. Regarding the risk factors associated with infection, the HBV prevalence rate was higher among older participants, foreigners and those with lower formal educational level. Increasing age has been extensively described as an independent factor associated with HBV exposure^19^. The higher frequency detected among foreigners could be attributed to the lower level of vaccine coverage in their original countries (mostly from Latin America). However, the scarce data on previous immunization (anti-HBs) prevents from making an assertion about it. This association, together with the high prevalence rates observed among those with lower formal educational level, was described several years ago among men who have sex with men and female sex workers^3,7^.

In relation to *T. pallidum* prevalence, this study reveals that syphilis was present in less than 1% of the group, with the prevalence reported in previous studies being higher among at-risk groups (22.4% among female sex workers, 20.5% among men who have sex with men, and 50.4% among female transgender sex workers)^4,10,20,21^. Data from the Ministry of Health reveals that syphilis has been increasing over the past five years in the country. The increase among pregnant women and congenital syphilis individuals is especially concerning. Among pregnant women, the prevalence increased from 2% in 2013 to 3.2% in 2017. The rate of the reported congenital syphilis has subsequently increased from 1 case per 100,000 live births in 2013 to 1.7/100,000 in 2017. Although the syphilis prevalence found in our specific population is low, national surveillance data highlight the need for targeted syphilis prevention strategies focused on young individuals^22^.

With respect to regional differences, in this study we observed a higher frequency of *T. pallidum* in Buenos Aires and Santa Fe, compared to Cordoba; and a higher frequency of previous exposure to HBV in Buenos Aires compared to other cities. These results are in line with previous data published by the Ministry of Health, where syphilis incidence has been increasing in several areas over the past five years, but particularly in Buenos Aires^22^. Even when no specific information is available on HBV prevalence in different cities of the country, one of the reasons for the higher frequency in Buenos Aires can be the fact that some groups of the population with high HBV prevalence, like men who have sex with men and female transgender, are most frequently concentrated in bigger cities, constituting thus a source of transmission for others.

This study was aimed to assess an adult population without specific risk factors and without the underestimations that blood donors usually have. Our results demonstrate that even when prevalence rates of viral hepatitis were not alarming, premarital screening could constitute an instance of diagnosis, vaccination and inclusion in care for those in need. In fact, in some countries, this strategy has already been implemented or it is under consideration^23–26^. This information will allow the national hepatitis programs to design public policies in order to diminish the impact of these infections on the population.
